# Medical and economic consequences of perioperative complications in older hip fracture patients

**DOI:** 10.1007/s11657-020-00843-z

**Published:** 2020-11-06

**Authors:** Tom Knauf, Juliana Hack, Juliane Barthel, Daphne Eschbach, Carsten Schoeneberg, Steffen Ruchholtz, Benjamin Buecking, Rene Aigner

**Affiliations:** 1 Center for Orthopaedics and Trauma Surgery, University Hospital Giessen and Marburg GmbH, Baldingerstraße 1, 35043 Marburg, Germany; 2 Department of Orthopedic and Emergency Surgery, Alfried Krupp Hospital, Alfried-Krupp-Straße 21, 45131 Essen, Germany; 3 Center for Orthopaedics and Trauma Surgery, DRK-Kliniken Nordhessen, Hansteinstraße 29, 34121 Kassel, Germany

**Keywords:** Ortho-geriatrics, Geriatric fracture, Hip fracture, Treatment costs

## Abstract

***Summary*:**

Patients suffering from complications during inpatient treatment after hip fracture surgery are associated with a worse mid-term outcome. While surgically treatable complications only delay the healing process, internal complications seem to worsen the outcome in the long run. All complications come with significant increased costs during the hospital stay.

**Purpose:**

Due to the demographic changes, the importance of hip fractures is still increasing nowadays. Not only surgical but also medical complications represent a major challenge in the treatment of those patients. Nevertheless, only few is known about the functional, medical, and economic consequences of complications.

**Methods:**

A total of 402 hip fracture patients ≥ 60 years were observed prospectively at a German university hospital. Complications were assessed during the inpatient stay and classified by Clavien and Dindo. Afterwards their influence on acute care costs was examined as well as their influence on the mortality, health-related quality of life (HRQL) (EQ5D), functional capacities (Barthel index), and mobility (Tinetti score) in the follow-up periods of 6 and 12 months.

**Results:**

Complications that required surgical revision/treatment (type III) were associated with an increased 6 months’ mortality, while type II and IV complications did not influence mortality after 6 and 12 months. Six months after surgery, HRQL, Barthel score, and Tinetti score were reduced in patients suffering from all different types of complications. After 12 months however, HRQL, Barthel score, and Tinetti score following type II and IV complications remained reduced, while the scores improved in patients suffering from type III complication. All types of complications led to significantly increased acute care costs.

**Conclusions:**

The results of the present study emphasize the crucial role of perioperative complications in older patients with hip fractures. Therefore, special attention has to be given to the prevention of those complications, e.g., with orthogeriatric treatment models, which have been shown to be effective in the reduction of complications.

## Introduction

Due to the demographic changes, the treatment of geriatric patients is gaining more attention nowadays. Hip fractures represent typical fragility fractures and their incidence is still rising [1–3]. A worldwide increase in hip fractures from 1.7 million in 1990 to 6.3 million by 2050 has been estimated by Cooper et al. [4]. The co-existence of relevant co-morbidities, polypharmacy, cognitive impairment, and impaired mobility results in high rates of complications in this vulnerable population [5, 6].

Complication rates around 20–75% have been described in the literature with most of the complications being not directly associated with the surgical procedure [5, 7–12]. Surgical complications account for only 6.9%, mechanical failure and infection being the most common ones [7]. However, medical but not surgical complications have been shown to be associated with increased mortality [7, 13].

Previous studies focus on risk factors regarding peri-/postoperative complications in hip fracture patients. Several authors state number of comorbidities/American Society of Anesthesiologists (ASA)-Score [8, 11, 14], age [8, 11, 14], general anesthesia [12], delayed surgical treatment [9, 11, 12, 15], enteral steroids [8], frailty [14, 16], and male gender [8] as risk factors for complications in those patients. Some authors already pointed out the economic consequences [17, 18]. However, studies dealing with consequences of perioperative complications in patients with hip fractures are rare. Therefore, the aim of this study was to assess the influence of complications on mortality, health-related quality of life (HRQL) (EQ-5D), functional capabilities (Barthel Score), mobility (Tinetti score), and their economical acute care costs.

## Patients and methods

### Patients and treatment parameters

The present study is a sub-analysis of a prospective observational study including a total of 402 patients with hip fractures treated between April 2009 and October 2011. Patients ≥ 60 years with operative treatment due to hip fractures were included. Exclusion criteria were malignancy-related fractures and polytrauma patients (ISS ≥ 16 [19]). Five hundred thirty-nine patients with fracture were treated at our hospital during the observation period. Four hundred seventy-seven patients met the inclusion criteria. Of those 477 patients, 75 patients declined participation. We obtained approval by the local Ethics committee (AZ 175/08). Each patient or their legal guardians provided written informed consent for participation in the study. Mortality, clinical course, treatment costs, and short-term follow-up are already published in some publications of our study group [13, 20–22].

The following baseline data were documented from all patients: age, fracture classification (nondisplaced femoral neck fractures, displaced femoral neck fractures, stable pertrochanteric fractures, unstable pertrochanteric fractures, and subtrochanteric fractures), American Society of Anaesthesiologists (ASA) classification [23], Charlson Comorbidity Index [24], and Mini-Mental State Examination [25].

The following data were collected during time of hospitalization: time to surgery (≤ 24 h vs. > 24 h), surgical procedure (screw fixation, intramedullary nail, dynamic hip screw, hemiarthroplasty, and total hip arthroplasty), ICU readmission after surgery, transfusion of erythrocyte concentrates, and duration of stay at the acute-care hospital. Follow-up was obtained after 6 months and 1 year*.* EQ5D-5L [26], Barthel score [27], and Tinetti score [28] were collected during the different follow-up examinations. The Barthel Score (range 0–100 points) records essential everyday functions of the patients. Patients are divided into independent, partially independent, needy and largely dependent on care. The Tinetti score (range 0–28 points) is divided into 2 parts, a balance test and a walking test. The Tinetti score can be used to quantify the fall risk of the patient. Both tests are established screening tools in the treatment of geriatric patients. Complications were assessed during the inpatient stay and classified by Clavien and Dindo. The current classification is an update of the previous classification by Clavien in 1992. It was published in 2004 by Dindo et al. Grade 1 complication represents any deviation from the normal without the need of pharmacological treatment (except for antiemetics, antipyretics, analgetics, diuretics, electrolytes, and physiotherapy) or surgical, endoscopic, and radiological interventions. This includes for example transient elevation of the serum creatinine, atelectasis requiring physiotherapy, and noninfectious diarrhea. Grade 2 complication stands for a pharmacological treatment with drugs other than such allowed in grade 1, for example tachyarrhythmia requiring β-receptor antagonist, pneumonia, and urinary tract infections treated with antibiotics. Grade 3 complications require surgical, endoscopic, or radiological intervention like wound infection with the need of surgical intervention with the need of an anesthesia or pleural effusion with the need of a pleura drainage. Life-threatening complications are defined as grade 4 complications This includes for example acute renal failure requiring dialysis lung failure requiring intubation [29].

### Costs for acute inpatient treatment

The methodology regarding the calculation of the costs that has already been described in a prior study of our group [30]. All potential cost factors were recorded as accurately as possible for each of the 402 patients individually. Afterwards, the actual costs for each cost factor were retrospectively calculated with help of the controlling division in our financial department. For each patient, up to 196 individual parameters were recorded [30].

### Data management and analysis

Data were collected in a FileMaker® database (FileMaker Inc., Santa Clara, CA, USA). We performed double entry with a plausibility check to monitor data quality. IBM SPSS Statistics 24 (Statistical Package for the Social Sciences, IBM Corporation, Armonk, NY, USA) was used for statistical analysis. To identify possible influencing factors, bivariate analysis was conducted using Student’s *t* test or ANOVA. Statistical significance was assumed at *p* < 0.05. Finally, multivariate analysis with backward selection was performed to detect independent influencing factors (inclusion at *p* < 0.05, exclusion at *p* > 0.10).

## Results

A total of 402 patients (293 women and 109 men) with an average age of 81.3 years (95% confidence interval (CI) 80.5–82.1) were included in the study. Baseline characteristics are shown in Table 1.

**Table 1:** Baseline characteristics

**Age in years ± SD (min–max)**	81 ± 8 (60–99)
**Sex (*n*=)**	
Female	293 (73%)
Male	109 (37%)
**Fracture classification (*n*=)**	
Nondisplaced femoral neck fracture	24 (6%)
Displaced femoral neck fracture	171 (43%)
Stable pertrochanteric fracture	65 (16%)
Unstable pertrochanteric fracture	121 (30%)
Subtrochanteric fracture	21 (5%)
**ASA Score ± SD (min–max)**	2.9 ± 0.6 (1–5)
**Charlson Comorbidity Index ± SD (min–max)**	2.4 ± 2.3 (0–12)
**MMSE ± SD (min–max)**	20 ± 9 (0–30)
**Time between hospital admission and surgery in hours ± SD (min–max)**	18 ± 13 (1–92)
**Surgical procedure**	
Screw fixation (dynamic hip screw, cannulated screws)	27 (7%)
Intramedullary nail	210 (52%)
Hemiarthroplasty	143 (36%)
Total hip arthroplasty	22 (6%)
**Length of stay in days ± SD (min–max)**	14 ± 6 (2–50)
**Most common complications (*n*=)**	
Type II	110
1. Urinary tract infection	92
2. Pneumonia	7
3. Newly occurred atrial fibrillation	3
Type III	47
1. Hematoma/seroma	19
2. Pleural effusion	12
3. Wound infection	5
Type IV	21
1. Myocardial infarction	10
2. Acute renal failure requiring dialysis	3
3. Stroke	3

*SD* standard deviation, *ASA* American Society of Anesthesiologists, *MMSE* Mini-Mental Status Examination

### Influence of complications on mortality

The mortality of patients without complications was 13.7% in the 6 months’ follow-up and 22.3% in the 12 months’ follow-up. Patients suffering from type III complications had a significant higher mortality (27.7%; *p* = 0.018) after 6 months compared to patients not suffering from any of these complications. In the 12 months’ follow-up, this trend could be detected as well (35.9%), however without statistical significance (*p* = 0.072). Complications type II and IV according to Clavien and Dindo showed no significant influence on mortality after 6 or 12 months (Table 2).

**Table 2:** Mortality

Complication (Clavien and Dindo)	Mortality 6 months % (n=)	*p* value*	Mortality 12 months % (*n*=)	*p* value*
No complication	13.7% (31)		22.3% (44)	
II	20.0% (22)	0.138	29% (27)	0.216
III	27.7% (13)	0.018	35.9% (14)	0.072
IV	15.0% (3)	0.873	27.8% (5)	0.598

^*^Compared to no complication

### Influence of complications on HRQL (EQ-5D)

Type II (0.56 (min. − 0.2; max. 1.0); *p* = 0.032) as well as type III (0.46 (min. 0.0; max. 1.0); *p* = 0.010) and type IV (0.41 (min. 0.0; max. 0.9); *p* = 0.005) complications were associated with a significant decrease of EQ-5D after 6 months compared to no complications (0.65 (min. − 0.2; max. 1.0)). However, after 12 months, only patients suffering from type IV complications (0.42 (min. 0.1; max. 0.9); *p* = 0.017) had a significantly lower EQ-5D compared to patients not suffering from any complication (0.66 (min. − 0.2; max. 1.0). A trend towards a reduced EQ-5D after 12 months was detected in patients suffering from type II complications; however, this was not statistically significant (*p* = 0.051). No impact of type III complications was found after 12 months (Table 3).

**Table 3:** EQ-5D

Complication (Clavien and Dindo)	EQ-5D 6 months Mean (95%CI)	*p* value*	EQ-5D 12 months Mean (95%CI)	*p* value *
No complication	0.65 (0.60–0.69)		0.66 (0.61–0.71)	
II	0.56 (0.48–0.63)	*p* = 0.032	0.56 (0.46–0.65)	*p* = 0.051
III	0.46 (0.33–0.59)	*p* = 0.010	0.56 (0.38–0.74)	*p* = 0.332
IV	0.41 (0.26–0.60)	*p* = 0.005	0.42 (0.25–0.59)	*p* = 0.017

*CI* confidence interval

^*^Compared to no complication

### Influence of complications on functional capabilities (Barthel score)

Patients not suffering from a complication had a Barthel score of 75.4 (min. 0; max. 100) after 6 months and 75.6 (min. 0; max. 100) after 12 months. Patients suffering from type II complications during hospitalization had a significantly lower Barthel score after 6 months (61.3 (min. 0; max. 100); *p* = 0.001) and 12 months (58.7 (min. 0; max. 100); *p* = 0.001). Similar results were found in patients who suffered from type IV complications after 6 months (41.7 (min. 5; max. 100); *p* < 0.001) and 12 months (50.8 (min. 5; max. 100); *p* = 0.013). Patients suffering from type III complications showed a trend to a lower Barthel score after 6 months (62.7 (min. 10; max. 100); *p* = 0.075), while after 12 months the Barthel score increased to (72.6 (min. 15; max. 100); *p* 0.770) (Table 4).

**Table 4:** Barthel score

Complication (Clavien and Dindo)	Barthel score 6 months Mean (95%CI)	*p* value*	Barthel score 12 months Mean (95%CI)	*p* value *
No Complication	75.4 (71.4–79.5)		75.6 (70.6–80.6)	
II	61.3 (53.7–69.0)	*p* = 0.001	58.7 (49.2–68.2)	*p* = 0.001
III	62.7 (49.6–75.8)	*p* = 0.075	72.6 (57.9–87.4)	*p* = 0.770
IV	41.7 (25.1–58.2)	*p* < 0.001	50.8 (29.7–71.8)	*p* = 0.013

*CI* confidence interval

^*^Compared to no complication

### Influence of complications on mobility (Tinetti score)

Complications types II–IV (type II, 14.1 (min. 1; max. 28); *p* = 0.006; type III, 13.3 (min. 1; max. 28); *p* = 0.043; type IV, 9.3 (min. 0; max. 28); *p* = 0.002) showed a significant reduction in the Tinetti score after 6 months compared to no complication (17.4 (min. 1; max. 28)). Twelve months after the fracture, type II (14.0 (min. 1; max. 28); *p* = 0.002) and type IV complications (8.9 (min. 1; max. 23); *p* = 0.002) were associated with a significantly lower Tinetti score compared to patients not suffering from any of these complications (18.3 (min. 0; max. 28)). No significant influence was detected in patients with type III complications (Table 5).

**Table 5:** Tinetti score

Complication (Clavien and Dindo)	Tinetti score 6 months Mean (95%CI)	*p*-Value*	Tinetti score 12 months Mean (95%CI)	*p*-Value*
No complication	17.4 (16.1–18.8)		18.3 (16.7–19.9)	
II	14.1 (12.1–16.1)	*p* = 0.006	14.0 (11.4–16.5)	*p* = 0.002
III	13.3 (9.5–17.1)	*p* = 0.043	17.4 (12.7–22.0)	*p* = 0.689
IV	9.3 (4.2–14.4)	*p* = 0.002	8.9 (2.8–15.0)	*p* = 0.002
Complication (Clavien and Dindo)	6 months	*p* value *	12 months	*p* value *
No Complication				
II		*p* = 0.523		*p* = 0.010
III		*p* = 0.362		*p* = 0.327
IV		*p* = 0.875		*p* = 0.001

*CI*- Confidence Interval

^*^Compared to no complication

### Multivariate analysis

After adjusting for age, gender, and ASA score, a trend towards an increased mortality after 6 months was seen after type III complications (*p* = 0.066), whereas other types of complications did not show associations. HRQL was significantly reduced after type II–IV complications at 6 months’ follow-up (*p* values: type II, 0.046; type III, 0.010; type IV, 0.010). After 12 months, a trend towards reduced HRQL was seen after type II (*p* = 0.069), no association after type III and a significantly reduced HRQL after type IV complications (0.019). Type II and IV complications were significantly associated with a reduced Barthel score and Tinetti score after 6 and 12 month (*p* values: Barthel score: type II, 6 months, 0.001; 12 month, 0.001; type IV 6 months, < 0.001; 12 months, 0.010; Tinetti: type II 6 months, 0.005; 12 months, 0.010; type IV 6 months, 0.001; 12 months, 0.002). A trend towards reduced Barthel and Tinetti scores was seen after type III complications after 6 months (*p* values: BI, 0.058; Tinetti: *p* = 0.070), while after 12 months no more associations were observed.

### Treatment costs: distribution and influence of complications

**Fig. 1:**
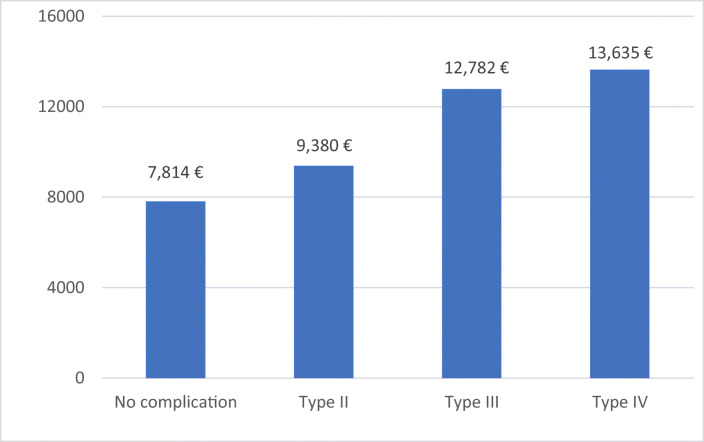
Costs depending on the Clavien and Dindo classification

The average costs for overall acute-care treatment were 8853€. All types of complications resulted in significantly higher treatment costs. Acute care costs of patients suffering from no complications averaged 7814€ (95%CI 7291–8338 €). A significant increase was noted in patients suffering from type II complication (9380€; (95%CI 8192–10,569 €) *p* = 0.004), type III complications (12,782€; (95%CI 10,014–15,550 €) *p* < 0.001), or type IV complications (13,635€; (95%CI 9400–17,870 €) *p* < 0.001) (Fig. 1).

## Discussion

The aims of this study were to evaluate the influence of complications in older patients with hip fractures on mortality, health-related quality of life (HRQL) (EQ-5D), functional capabilities (Barthel score), mobility (Tinetti score), and their economical acute care costs.

### Influence of complications on mortality

Several authors investigated mortality after hip fractures in older patients. Most of these studies focused on patients’ characteristics and data from hospitalization. Usually postoperative complications were not investigated. ASA-Score [31, 32], comorbidities [31, 33], duration of postoperative immobilization [31], gender [32–34], age [32, 33], and surgical delay [32] have been shown to significantly increase mortality after hip fracture. In this study, predominantly type III complications increased mortality. This underlines that mainly those complications requiring surgical revision seem to increase the risk of dying in a 12-month follow-up after surgical fixation of hip fractures.

### Influence of complications on HRQL

Although several studies investigated the influence of hip fractures on quality of life, to the best of our knowledge few have included the influence of complications. Alexiou et al. showed in a review that the majority of those patients did not regain their pre-fracture quality of life [35]. Reviewing 49 studies, they stated comorbidities, female gender, and undernutrition as factors associated with a negative impact on health-related quality of life. These factors also lead to longer duration of hospital stay, severe postoperative pain, and complications [35]. Taking a closer look at the occurrence of complications, all types resulted in a significantly lower EQ-5D 6 months after the fracture. No statistically significant difference of HRQL was found between patients suffering from type III complications and those not suffering from any complication at 12 months’ follow-up. In contrast, HRQL in patients with type II or IV complications does not equalize during the 12 months follow up. It appears that patients suffering from type III complications experience notable reduction of HRQL in the short term; however, they show a high potential of recovery. In contrast, no further improvement between 6 and 12 months was seen in patients who have suffered from a type II or IV complication. This suggests that type II and IV complications tend to reflect a worsening of the general condition and an increase in secondary diseases, while typical type III complications, such as surgical revision, do not necessarily reflect the patient’s state of health. Hansson et al. showed in their retrospective cohort study similar results concerning patients suffering from complications. They found that the absence of complications was associated with higher satisfaction and lower pain, even 6 months after surgery [36].

### Influence of complications on functional capabilities

Patients suffering from type II and type IV complications had a significantly lower Barthel score 6 and 12 months after surgery. Patients suffering from type III complications showed a trend to a lower Barthel score 6 months after surgery, which was not significant. However, they almost regained their functional capabilities after 12 months compared to patients without any complications. The additional surgery seems to prolong functional recovery of those patients, but they are not significantly affected at the 1-year follow-up. In contrast, type II and type IV complications may represent a surrogate parameter for the frailty of the patients like mentioned already above. Several authors investigated functional outcome after hip fracture. Most of them focused on patients’ characteristics and data from hospitalization but did not examine the influence of different types of complications on the mid-term outcome. In the recent literature, inconsistent results have been published by different authors. This might be due to the different study collectives and study protocols. Patient characteristics that have been associated with poor functional outcome by many authors are increased age [37–40], poor mental status [37, 39, 41, 42], and higher degree of comorbidity [38]. While there are now contradictory statements about high age alone as a risk factor, there is agreement about the influence of the previous state of health [43].

### Influence of complications on mobility

“Tinetti score” was used to illustrate the influence of complications on mobility after hip fracture. All types of complications lead to a significantly lower Tinetti score compared to patients not suffering from complications 6 months after the surgery. Similar to HRQL and functional status patients with type III complications improved after 12 months, whereas type II and IV complications were associated with a significantly reduced Tinetti score also after 12 months. Again, a possible explanation could be that patients suffering from surgical complications require a couple more months for rehabilitation. Nevertheless, once survived patients suffering from type III complications achieve comparable mobility to patients without complications whereas mobility in patients with type II and IV complications remains significantly decreased. Again, these complications seem to be a surrogate parameter for an overall poor general health status, which could be reasonable for reduced mobility.

### Economical acute care consequences

Our group already showed that ASA-Score, Charlson Comorbidity Index, and fracture location are influencing factors that lead to increased costs of acute care for hip fracture treatment [30]. Complications during in-hospital treatment do not only have an impact on patient’s mobility, mortality, functional capability, and health-related quality of life; they go along with significantly increased acute care costs as well. Broderick et al. showed in their selective review regarding failure of geriatric fractures that implant failure does not only lead to a twofold increase in length of stay, but leads to a doubling of healthcare costs as well [44]. Other authors showed increased costs after implant failure, wound infection, others reasons of reoperations, and medical complications [17, 18, 44, 45]. In line with these findings, type III complications were associated with increased costs in the present study. However, patients who needed surgical revision were not those causing the highest costs in our study sample. Patients suffering from type IV complications were associated with the highest healthcare costs. This might be due to prolonged intensive care treatment and the necessary intensive inpatient treatment after suffering from these complications (e.g., dialysis). As expected, type III complications increase healthcare costs in a significant amount. Even type II complications increase healthcare costs during inpatient treatment significantly. This is explained by an extra amount of medical or personal capacities (e.g., drugs like antibiotics) required by patients suffering from type II complications.

### Strengths and limitations

The results of the present study are limited by some factors. We included only patients from one single center, and only selected parameters were collected and analyzed. The fact that orthogeriatric treatment, which has been proven to be effective in reducing complications, was not performed in the present analysis represents another limitation. Furthermore, a cost analysis, regardless of how detailed and accurate it is, contains some assumptions. For example, it is not possible to exactly determine the duration of physician or nurse-patient contact during the acute ward phase. Another drawback of this study is that it only includes primary hospitalization costs. Finally, the age restriction ≥ 60 years has to be mentioned as a limitation, as this is not the typical definition of a geriatric patient and therefore non-geriatric patients may have been included.

Nevertheless, the prospective design and its large patient collective are strengths of this study. Furthermore, the cost analysis represents the result of an extremely detailed analysis of the actual costs of care for every individual patient.

## Conclusion

Complications after hip fracture treatment do not only increase acute care costs, but also represent a burden for those patients during the following rehabilitation. Complications that require a surgical revision/ treatment (type III) increase acute care costs as well as 6-month mortality significantly. However, once survived, type III complications are not associated with reduced HRQL, decreased functional capacities, or impaired mobility after 12 months. Nevertheless, rehabilitation seems delayed as these patients show reduced HRQL, functional capacities, and mobility after 6 months.

In contrast, type II and type IV complications are not associated with increased mortality after 6 and 12 months. However, most probably being a surrogate parameter for frailty, they are associated with reduced HRQL, reduced functional outcomes, and impaired mobility after 6 and 12 months. The results of the present study emphasize the crucial role of perioperative complications in geriatric patients with hip fractures from a medical as well as from an economic perspective. Therefore, special attention has to be given to the prevention of those complications.

## Data Availability

Not applicable.

## References

[1] Bundesamt S Krankenhauspatienten: Deutschland, Jahre, Geschlecht, Altersgruppen, Hauptdiagnose ICD-10 (1–3-Steller Hierarchie)

[2] Marks R (2010) Hip fracture epidemiological trends, outcomes, and risk factors, 1970–2009. Int J Gen Med PMC2866546 20463818

[3] Icks A, Arend W, Becker C, Rapp K, Jungbluth P, Haastert B (2013) Incidence of hip fractures in Germany, 1995-2010. Arch Osteoporos 8. 10.1007/s11657-013-0140-5 10.1007/s11657-013-0140-5 23674147

[4] Cooper C, Campion G, Melton LJ (1992). Hip fractures in the elderly: a world-wide projection. Osteoporos Int.

[5] Carpintero P, Caeiro JR, Carpintero R, Morales A, Silva S, Mesa M (2014). Complications of hip fractures: a review. World J Orthop.

[6] Tosounidis TH, Castillo R, Kanakaris NK, Giannoudis PV (2015). Common complications in hip fracture surgery: tips/tricks and solutions to avoid them. Injury..

[7] Tsang STJ, Aitken SA, Golay SK, Silverwood RK, Biant LC (2014). When does hip fracture surgery fail?. Injury..

[8] Roche JJW, Wenn RT, Sahota O, Moran CG (2005). Effect of comorbidities and postoperative complications on mortality after hip fracture in elderly people: prospective observational cohort study. Br Med J.

[9] Sircar P, Godkar D, Mahgerefteh S, Chambers K, Niranjan S, Cucco R (2007). Morbidity and mortality among patients with hip fractures surgically repaired within and after 48 hours. Am J Ther.

[10] Schmid S, Blobner M, Haas B, Lucke M, Neumaier M, Anetsberger A, Jungwirth B (2019). Perioperative multi-system optimization protocol in elderly hip fracture patients: a randomized-controlled trial. Can J Anesth.

[11] Poh KS, Lingaraj K (2013). Complications and their risk factors following hip fracture surgery. J Orthop Surg (Hong Kong).

[12] Flikweert ER, Wendt KW, Diercks RL, Izaks GJ, Landsheer D, Stevens M, Reininga IHF (2018). Complications after hip fracture surgery: are they preventable?. Eur J Trauma Emerg Surg.

[13] Knauf T, Bücking B, Bargello M, Ploch S, Bliemel C, Knobe M, Ruchholtz S, Eschbach D (2019). Predictors of long-term survival after hip fractures?—5-year results of a prospective study in Germany. Arch Osteoporos.

[14] Folbert EC, Hegeman JH, Gierveld R, van Netten JJ, Velde D, ten Duis HJ, Slaets JP (2017). Complications during hospitalization and risk factors in elderly patients with hip fracture following integrated orthogeriatric treatment. Arch Orthop Trauma Surg.

[15] Al-Ani AN, Samuelsson B, Tidermark J (2008). Early operation on patients with a hip fracture improved the ability to return to independent living: a prospective study of 850 patients. J Bone Jt Surg - Ser A.

[16] Kistler EA, Nicholas JA, Kates SL, Friedman SM (2015). Frailty and short-term outcomes in patients with hip fracture. Geriatr Orthop Surg Rehabil.

[17] Pollard TCB, Newman JE, Barlow NJ, Price JD, Willett KM (2006). Deep wound infection after proximal femoral fracture: consequences and costs. J Hosp Infect.

[18] Lüthje P, Helkamaa T, Nurmi-Lüthje I, Kaukonen JP, Kataja M (2014). An 8-year follow-up study of 221 consecutive hip fracture patients in Finland: analysis of reoperations and their direct medical costs. Scand J Surg.

[19] Baker SP, O’Neill B, Haddon W, Long WB (1974). The injury severity score: a method for describing patients with multiple injuries and evaluating emergency care. J Trauma.

[20] Knauf T, Buecking B, Hack J, Barthel J, Bliemel C, Aigner R, Ruchholtz S, Eschbach D (2019). Development of the Barthel Index 5 years after hip fracture: results of a prospective study. Geriatr Gerontol Int.

[21] Eschbach D, Bliemel C, Oberkircher L, Aigner R, Hack J, Bockmann B, Ruchholtz S, Buecking B (2016). One-year outcome of geriatric hip-fracture patients following prolonged ICU treatment. Biomed Res Int.

[22] Aigner R, Hack J, Eschbach D, Ruchholtz S, Knobe M, Dodel R, Buecking B (2018). Is treatment of geriatric hip fracture patients cost-covering? Results of a prospective study conducted at a German University Hospital. Arch Orthop Trauma Surg.

[23] Carow J, Carow JB, Coburn M, Kim BS, Bücking B, Bliemel C, Bollheimer LC, Werner CJ, Bach JP, Knobe M (2017). Mortality and cardiorespiratory complications in trochanteric femoral fractures: a ten year retrospective analysis. Int Orthop.

[24] Charlson ME, Pompei P, Ales KL (1987) A new method of classifying prognostic comorbidity in longitudinal studies: development and validation 10.1016/0021-9681(87)90171-8: Journal of Chronic Diseases | ScienceDirect.com. J Chronic 10.1016/0021-9681(87)90171-8 3558716

[25] Folstein M, Folstein S (1975) A practical method for grading the cognitive state of patients for the clinician. J Psychiatr Res 10.1016/0022-3956(75)90026-6 1202204

[26] (2009) EQ5D - A standardised instrument for use of as a measure of health outcome- User Guide. https://euroqol.org/

[27] Lübke N, Meinck M, Von Renteln-Kruse W (2004). The Barthel Index in geriatrics. A context analysis for the Hamburg Classification Manual. Z Gerontol Geriatr.

[28] Tinetti ME (1986). Performance-oriented assessment of mobility problems in elderly patients. J Am Geriatr Soc.

[29] Dindo D, Demartines N, Clavien PA (2004). Classification of surgical complications: a new proposal with evaluation in a cohort of 6336 patients and results of a survey. Ann Surg.

[30] Aigner R, Meier Fedeler T, Eschbach D, Hack J, Bliemel C, Ruchholtz S, Bücking B (2016). Patient factors associated with increased acute care costs of hip fractures: a detailed analysis of 402 patients. Arch Osteoporos.

[31] Camurcu Y, Cobden A, Sofu H, Saklavci N, Kis M (2017). What are the determinants of mortality after cemented bipolar hemiarthroplasty for unstable intertrochanteric fractures in elderly patients?. J Arthroplast.

[32] Daugaard CL, Jørgensen HL, Riis T, et al (2012) Is mortality after hip fracture associated with surgical delay or admission during weekends and public holidays? Acta Orthop 10.3109/17453674.2012.747926 PMC3555458 23140106

[33] Geiger F, Zimmermann-Stenzel M, Heisel C, Lehner B, Daecke W (2007). Trochanteric fractures in the elderly: the influence of primary hip arthroplasty on 1-year mortality. Arch Orthop Trauma Surg.

[34] Omsland TK, Emaus N, Tell GS, Magnus JH, Ahmed LA, Holvik K, Center J, Forsmo S, Gjesdal CG, Schei B, Vestergaard P, Eisman JA, Falch JA, Tverdal A, Søgaard AJ, Meyer HE (2014). Mortality following the first hip fracture in Norwegian women and men (1999-2008). A NOREPOS study. Bone.

[35] Alexiou KI, Roushias A, Evaritimidis S, Malizos KN (2018) Quality of life and psychological consequences in elderly patients after a hip fracture: a review. Clin Interv Aging 10.2147/CIA.S150067 PMC5790076 29416322

[36] Hansson S, Rolfson O, Åkesson K, et al (2015) Complications and patient-reported outcome after hip fracture. A consecutive annual cohort study of 664 patients. Injury. 10.1016/j.injury.2015.07.024 10.1016/j.injury.2015.07.024 26298023

[37] Ishidou Y, Koriyama C, Kakoi H, Setoguchi T, Nagano S, Hirotsu M, Yamamoto T, Yokouchi M, Komiya S (2017). Predictive factors of mortality and deterioration in performance of activities of daily living after hip fracture surgery in Kagoshima, Japan. Geriatr Gerontol Int.

[38] Vergara I, Vrotsou K, Orive M, Gonzalez N, Garcia S, Quintana JM (2014) Factors related to functional prognosis in elderly patients after accidental hip fractures: a prospective cohort study. BMC Geriatr 10.1186/1471-2318-14-124 PMC4280690 25425462

[39] Alegre-López J, Cordero-Guevara J, Alonso-Valdivielso JL, Fernández-Melón J (2005). Factors associated with mortality and functional disability after hip fracture: an inception cohort study. Osteoporos Int.

[40] Haentjens P, Autier P, Barette M, Boonen S (2005). Predictors of functional outcome following intracapsular hip fracture in elderly women: a one-year prospective cohort study. Injury..

[41] Benedetti MG, Ginex V, Mariani E, et al (2015) Cognitive impairment is a negative short-term and long-term prognostic factor in elderly patients with hip fracture. Eur J Phys Rehabil Med 25998064

[42] Tarazona-Santabalbina FJ, Belenguer-Varea Á, Rovira Daudi E, Salcedo Mahiques E, Cuesta Peredó D, Doménech-Pascual JR, Gac Espínola H, Avellana Zaragoza JA (2015). Severity of cognitive impairment as a prognostic factor for mortality and functional recovery of geriatric patients with hip fracture. Geriatr Gerontol Int.

[43] Eschbach DA, Oberkircher L, Bliemel C, Mohr J, Ruchholtz S, Buecking B (2013). Increased age is not associated with higher incidence of complications, longer stay in acute care hospital and in hospital mortality in geriatric hip fracture patients. Maturitas.

[44] Broderick JM, Bruce-Brand R, Stanley E, Mulhall KJ (2013) Osteoporotic hip fractures: the burden of fixation failure. Sci World J 10.1155/2013/515197 PMC3580900 23476139

[45] Liu R, Chao A, Wang K, Wu J (2018). Incidence and risk factors of medical complications and direct medical costs after osteoporotic fracture among patients in China. Arch Osteoporos.

